# Changes in total plasma and serum N-glycome composition and patient-controlled analgesia after major abdominal surgery

**DOI:** 10.1038/srep31234

**Published:** 2016-08-09

**Authors:** Ivan Gudelj, Marco Baciarello, Ivo Ugrina, Manuela De Gregori, Valerio Napolioni, Pablo M. Ingelmo, Dario Bugada, Simona De Gregori, Lovorka Đerek, Maja Pučić-Baković, Mislav Novokmet, Olga Gornik, Gloria Saccani Jotti, Tiziana Meschi, Gordan Lauc, Massimo Allegri

**Affiliations:** 1 Genos Glycoscience Research Laboratory, Zagreb, Croatia; 2 Department of Anesthesia, ICU and Pain Therapy, University Hospital of Parma, Parma, Italy; 3 SIMPAR Group (Study in Multidisciplinary Pain Research), Parma, Italy; 4 Department of Surgical Sciences, University of Parma, Parma, Italy; 5 University of Zagreb Faculty of Pharmacy and Biochemistry, Zagreb, Croatia; 6 Pain Therapy Service, Fondazione IRCCS Policlinico S. Matteo, Pavia, Italy; 7 YAP (Young Against Pain) group, Parma, Italy; 8 Department of Neurology and Neurological Sciences, Stanford University School of Medicine, Palo Alto, CA, USA; 9 Department of Anesthesia, Montreal Children’s Hospital, Canada; 10 Clinical and Experimental Pharmacokinetics Unit, Fondazione IRCCS Policlinico San Matteo, Pavia, Italy; 11 Department of Medical Biochemistry and Laboratory Medicine, Clinical Hospital Merkur, Zagreb, Croatia; 12 Department of Biomedical, Biotechnological and Translational Science (S.Bi.Bi.T.), University of Parma, Parma, Italy; 13 Department of Clinical and Experimental Medicine, University of Parma, Italy

## Abstract

Systemic inflammation participates to the complex healing process occurring after major surgery, thus directly affecting the surgical outcome and patient recovery. Total plasma N-glycome might be an indicator of inflammation after major surgery, as well as an anti-inflammatory therapy response marker, since protein glycosylation plays an essential role in the inflammatory cascade. Therefore, we assessed the effects of surgery on the total plasma N-glycome and the association with self-administration of postoperative morphine in two cohorts of patients that underwent major abdominal surgery. We found that plasma N-glycome undergoes significant changes one day after surgery and intensifies one day later, thus indicating a systemic physiological response. In particular, we observed the increase of bisialylated biantennary glycan, A2G2S[3,6]2, 12 hours after surgery, which progressively increased until 48 postoperative hours. Most changes occurred 24 hours after surgery with the decrease of most core-fucosylated biantennary structures, as well as the increase in sialylated tetraantennary and FA3G3S[3,3,3]3 structures. Moreover, we observed a progressive increase of sialylated triantennary and tetraantennary structures two days after surgery, with a concomitant decrease of the structures containing bisecting *N*-acetylglucosamine along with bi- and trisialylated triantennary glycans. We did not find any statistically significant association between morphine consumption and plasma N-glycome.

Glycosylation is an essential post-translational modification affecting the structure and the function of thousands of proteins^1^. The functional importance of glycosylation stems from the fact that glycans are integral parts of protein structure, but contrary to polypeptide parts, which are defined by the corresponding gene nucleotide sequences, glycan structures are versatile and responsive to environmental stimuli^2^,^3^. For more than 30 years it has been known that glycans undergo significant changes during pathological states^4^. Indeed, a recent comprehensive report endorsed by the US National Academies stated “*glycans are directly involved in the pathophysiology of every major disease*” and that “*additional knowledge from glycoscience will be needed to realize the goals of personalized medicine and to take advantage of the substantial investments in human genome and proteome research and its impact on human health*”^5^. Notably, glycans seem to play a particularly important role in the immune system as inter-individual variation in glycosylation may affect immune responses at multiple levels^6^ and their biomarker potential has been recognized^7^,^8^.

Major surgery provokes a vigorous inflammatory response with important clinical implications^9^,^10^. However, the factors influencing incidence and severity of clinical outcomes associated with the inflammatory response (including life-threatening perioperative complications), are currently not well understood, even though the connection between inflammation and pain has already been investigated^10^ and inflammatory background may even influence the response to therapies^11^. Furthermore, the level of perceived pain varies significantly between individuals undergoing very similar surgery^12^ and genetics may be helpful in predicting postoperative pain and morphine consumption^13^,^14^,^15^. Recent studies revealed the existence of a significant inter-individual variability in both total plasma and IgG glycome composition^16^,^17^, which turned out to be associated with several pathological states^18^,^19^,^20^,^21^.

Whether simultaneous changes in inflammatory response pain and glycome composition have a common genetic background is not known; arguably, higher pain is associated with higher systemic inflammation and whole glycome changes, and the existence of a shared genetic component is conceivable. In the present work we studied the effects of surgery on protein glycosylation and its potential association with self-administration of morphine in the postoperative period by evaluating total plasma N-glycome in two cohorts of patients undergoing major abdominal surgery and in a replication cohort.

## Results

Two cohorts of patients who underwent major abdominal surgeries were studied. In the discovery cohort, we analyzed total plasma N-glycome of 182 patients (104 males and 78 females) from two hospitals at five time points: intraoperative and 6, 12, 24, 48 hours after the surgery. The patients underwent either upper abdominal, lower abdominal, xiphopubic or bilateral subcostal surgery. They also self-regulated pain by administrating morphine. Morphine and its main metabolites (morphine-3-glucuronide and morphine-6-glucuronide) were measured by using reversed phase HPLC coupled with mass spectrometry (HPLC-MS/MS), based on sample deproteinization by the addition of acetonitrile as previously described^13^. Basic descriptives of this cohort are reported in Table 1.

**Figure 1:**
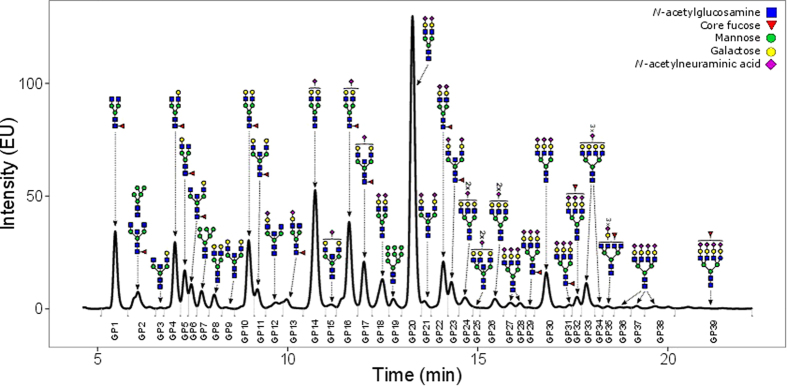
Representative chromatogram of 2-AB labeled N-linked glycans released from the plasma proteins and separated by HILIC-UPLC. The integration areas, together with a major structure presented in each glycan group are given. Glycan groups are numbered from GP1-GP39, as used in the paper.

No changes in the composition of plasma N-glycome occurred during the first six hours after the surgery (see Fig. 1, Supplementary Table S1), beside a statistically significant increase in biantennary glycans and a decrease in the sialylation of triantennary and tetraantennary structures (see Supplementary Table S2). A statistically significant increase in the most abundant structure of human plasma N-glycome, (bisialylated biantennary glycan, A2G2S[3,6]2; GP20) was observed 12 hours after surgery. This was the only structure increasing during this period, and it showed a progressive increase for 48 hours after the surgery. Concomitantly, the amount of non-fucosylated sialylated triantennary glycans A3G3S[3,6]2 (GP24), A3G3S[3,3]2 (GP26) and A3G3S[3,3,3]3 (GP27) decreased.

**Figure 2:**
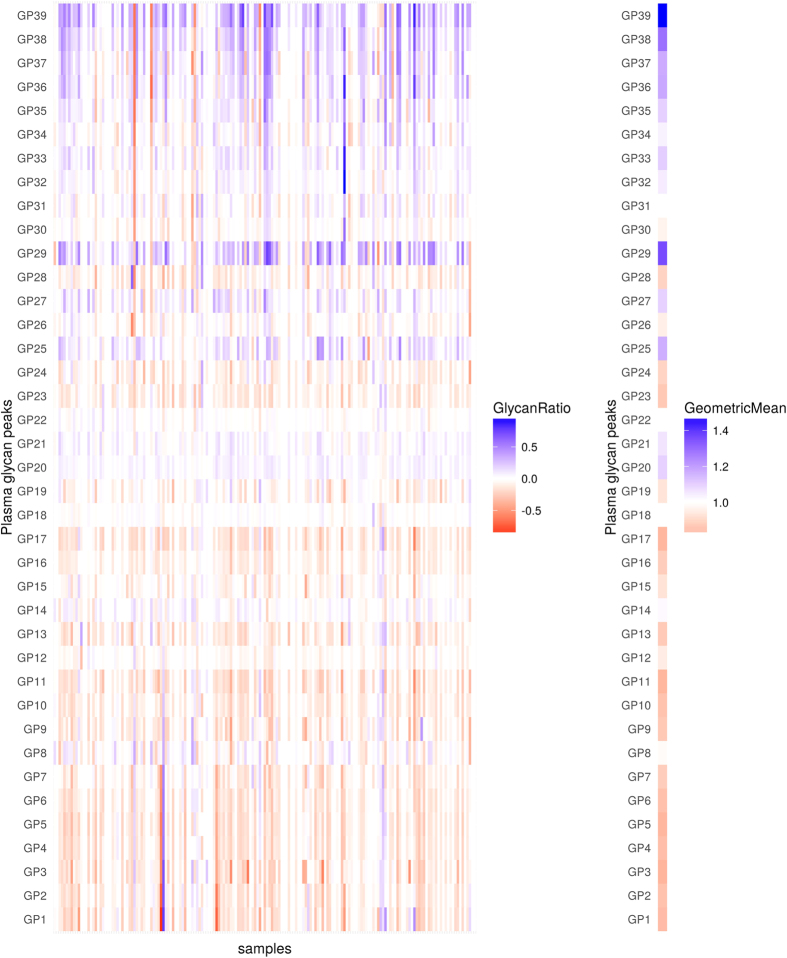
Changes in levels (abundance) of plasma glycans between day 0 and day 2 for the discovery cohort. Changes are presented through the logarithm of relative changes log_10_ (day2/day0) in the left plot and the geometric mean (as a measure of trends) of relative changes (day2/day0) in the right plot. The red color indicates decrease in glycan level and blue color indicates increase in glycan level; intensity of colors indicates the intensity of the changes. All changes are presented with respect to levels before the surgery.

Most significant changes were observed 24 hours after the surgery and were opposite compared to the ones occurring during the first six hours after surgery. In that period, the majority of core-fucosylated biantennary structures decreased, while sialylated tetraantennary and FA3G3S[3,3,3]3 (GP29) structures increased. Besides A2G2S[3,6]2 (GP20), a continuing increase of sialylated triantennary and tetraantennary structures could be noticed two days after surgery (Fig. 2, Table 2). In the same period all structures containing bisecting *N*-acetylglucosamine (GlcNAc) strongly decreased together with continuous decrease of bi- and trisialylated triantennary (A3G3S[3,6]2 (GP24) and A3G3S[3,3,6]3 (GP28)) glycans.

**Figure 3:**
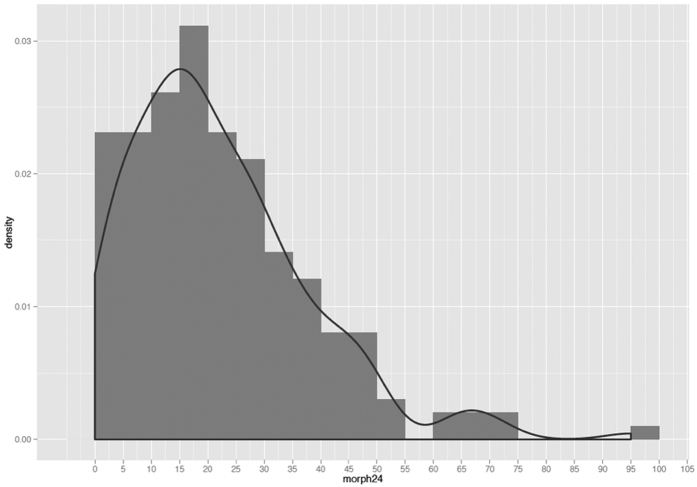
A quasi-Poisson distribution of morphine consumption.

Consumption of morphine showed a quasi-Poisson distribution (Fig. 3). We did not find any statistically significant associations in the discovery cohort between the postoperative PCA morphine consumption and plasma N-glycome. Multiple box plots showing glycoprotein levels at baseline and at 6-hour after the surgery by morphine consumption group (determined at 24-hours after the surgery) are reported in Supplementary Figs S1 and S2.

**Figure 4:**
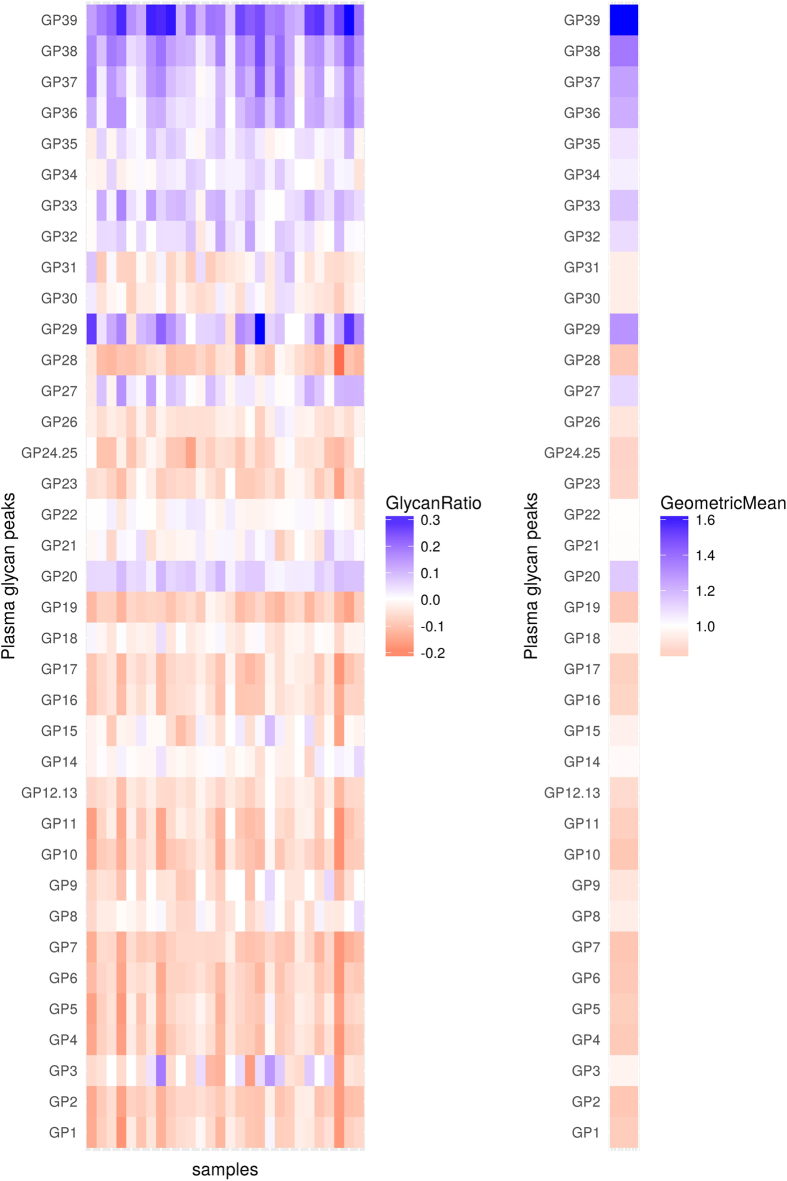
Changes in levels (abundance) of plasma glycans between day 0 and day 2 for the replication cohort. Changes are presented through the logarithm of relative changes log_10_ (day2/day0) in the left plot and the geometric mean (as a measure of trends) of relative changes (day2/day0) in the right plot. The red color indicates decrease in glycan level and blue color indicates increase in glycan level; intensity of colors indicates the intensity of the changes. All changes are presented with respect to levels before the surgery.

Given that similar changes in glycosylation can be seen in both plasma and serum^22^, for replication cohort we analyzed total serum N-glycomes of 28 patients. Basic descriptive information about this cohort is provided in Table 3. Sera samples were taken at three different time points: prior to surgery, and 24 and 48 hours after the surgery. Decrease of biantennary together with decrease of sialylated triantennary structures was presented one day after the surgery. At the same time, increase in sialylated tetraantennary and FA3G3S[3,3,3]3 (GP29) glycans, as well as in the most abundant structure in the human plasma N*-*glycome A2G2S[3,6]2 (GP20) could be seen (see Supplementary Table S3). Continuing decrease of biantennary and some of sialylated triantennary structures was observed two days after surgery (Fig. 4, Table 4). In the same period, the increase of triantennary and tetraantennary together with continuing increase of A2G2S[3,6]2 was noticed. Even though the majority of changes observed in the discovery study were observed also in the replication study (see Supplementary Table S4), the number of structures which showed decrease after 24 hours and increase after 48 hours was a little bit higher in the replication cohort, compared to the discovery cohort, which could be due to an artifact of increased penalization in multiple testing correction due to only two time points in the replication cohort compared to four time points in the discovery cohort.

## Discussion

In the present work we found that the plasma N-glycome undergoes significant changes after major abdominal surgery using two independent cohorts. Most of the changes occurred one day after the surgery and intensified one day later, thus indicating a systemic physiological response. The fact that only minor changes were observed six hours after surgery is not unexpected, since during this period the main inflammatory response is proximal to the place of surgery; which corresponds to a model of a biphasic inflammatory response after a gut surgery with activation of macrophages in the intestinal muscularis followed with leukocyte infiltration 24 hours after surgery^23^. Maximal changes in the plasma N-glycome coincide with the maximal increase in Interleukin 6 (IL6) expression, observed between 4 and 24 hours after surgery and remained elevated for up to 48–72 hours^24^,^25^,^26^,^27^,^28^. IL6 is believed to be an important regulator of plasma protein levels during inflammation^27^, and a recent GWAS study reported the association of single nucleotide polymorphisms in IL6 with IgG glycome composition^29^, indicating that this pro-inflammatory cytokine might be involved in the observed alterations of plasma N-glycome. A first limitation of our study is that we can not exactly rule out which correlation exists between systemic inflammatory response and glycome changes, and whether the same results could be retrieved in a different surgical cohort (receiving less invasive surgical procedures, likely to activate lower inflammatory response). Further studies should compare cytokines’ production with glycome variations in different types of surgical procedures (likely to be associated with different degrees of systemic inflammation).

**Figure 5:**
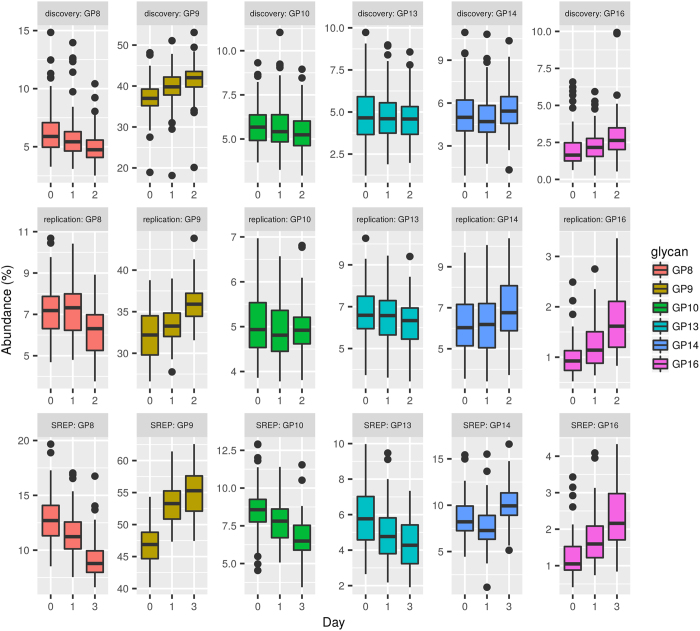
Graphical comparison of changes in glycan levels between the discovery cohort, the replication cohort and previously published data^20^ (SREP) as an additional replication of the results. The comparison is given through boxplots where the bottom and top of the box are the first and third quartiles, and the band inside the box is the second quartile (the median). Ends of the whiskers represent the lowest datum still within 1.5 IQR (interquartile range) of the lower quartile, and the highest datum still within 1.5 IQR of the upper quartile. Data beyond the end of the whiskers are outliers and plotted as points. Only a subset of directly comparable glycans is shown due to differences in measurement technique for the replication and discovery cohorts (Ultra-performance liquid chromatograph) and previously published data (High performance liquid chromatography). Statistical inference was not performed between cohorts.

When studying plasma glycome, it is important to acknowledge that variations in plasma protein concentrations are inseparable from changes in glycosylation of individual proteins. The human plasma N-glycome displays a significant inter-individual variability^16^, but within an individual it is temporally stable in homeostatic condition^30^. To alleviate the problem of inter-individual variation, for every patient we matched each post-operative sample to its relative pre-operatory sample as baseline control. Previously, we reported that the human plasma N-glycome can rapidly change when homeostasis is disrupted^20^ and this observation was confirmed in this study. A notable increase of triantennary and tetraantennary sialylated structures (all sialylated tetraantennary structures of the human plasma N-glycome with antennary fucose were presented in the group of structures which increased during inflammation) was observed within the first 24 hours after surgery and continued for the next 24 hours. These tetraantennary tetrasialylated structures with fucose on the outer arm are also known as the sialyl-Lewis X antigen, which has been already associated with inflammation^31^ and demonstrated to play an important role in leukocytes’ interaction with selectin ligands and initiation of inflammation^32^,^33^,^34^. Moreover, a strong decrease of all structures containing bisecting GlcNAc could be linked to the need of higher cell mobility during the inflammatory process. Indeed, bisecting GlcNAc confers unique lectin recognition properties to N*-*glycans and it obstructs other GlcNAc-trasferases that serve to form multiantennary sugar chains therefore leading to decrease in branch formation of N*-*glycans^35^. Since we observed increased branching during inflammation, the decrease of bisecting GlcNAc is not unexpected. Also, we noticed a slight decrease of sialylated tetraantennary along with an increase of biantennary structures six hours after surgery, which represents an opposite situation compared to what observed after 24 hours. This evidence is in line with our previous findings^20^ (Fig. 5), although the same declines and increases were observed a few hours later. This delay may be attributable to the different molecular mechanism underlying the inflammatory response after cardiac and abdominal surgeries.

We would like to investigate if the changes of morphine consumption could be affected also from the glycosylation of proteins as it could also affect bioavailability of the drug. Unfortunately, we did not find any statistically significant associations in the discovery cohort between the postoperative PCA morphine consumption and plasma N-glycome.

These results could be affected by the fact that the changes mentioned above cannot be exclusively attributed to the changes in glycosylation but also to the impact of changes in plasma protein composition. With the employed method we are not able to determine the main cause of the changes in the plasma N*-*glycome. After albumin, IgG is the most abundant protein in the human plasma and the most abundant glycoprotein^36^. The human IgG N*-*glycome is very well known^17^ as well as the influence of IL6 on its synthesis^25^. It is interesting to notice that IgG has been positively correlated with serum IL6 concentration during inflammation^25^, yet from our results it seems the IgG-linked N-glycans do not increase during inflammation. Moreover, we have observed a decrease of bianntenary N-glycan structures coming from IgG^17^,^36^. In addition, positive acute-phase proteins (APPs), such as α_1_-acid glycoprotein, α_1_-antitrypsin, hemopexin, vitronectin and ceruloplasmin are known to carry abundance of sialylated trianntenary and tetraantennary N-glycans^37^,^38^,^39^,^40^,^41^,^42^,^43^,^44^. The observed changes in those structures in our study probably originate from the changes in the levels of plasma proteins rather than changes in protein glycosylation. However, the situation is more complex with the most abundant structure in human plasma N-glycome, namely the A2G2S2; this structure can be found on the APPs decreasing during inflammation such as transferrin and α_2_-HS glycoprotein^44^,^45^,^46^,^47^, as well as on α_1_-acid glycoprotein, α_1_-antitrypsin, hemopexin, vitronectin, ceruloplasmin, haptoglobin, α_2_-macroglobulin and fibrinogen which increased^37^,^38^,^39^,^40^,^41^,^42^,^43^,^44^,^48^,^49^,^50^,^51^,^52^. Also, it is already known that glycosylation of some APPs changes during inflammation. For example, α_1_-acid glycoprotein antennary fucosylation increases during inflammation thus making the sialyl-Lewis X structure^37^,^53^,^54^,^55^,^56^, which increase was also noticed in our studies. Moreover, the same changes have been noticed on α_1_-antichymotrypsin and haptoglobin^31^. Since existing biomarkers of inflammation are discrete molecular species (APPs and inflammatory cytokines), we believe that total plasma N-glycan analysis shows an overall picture of changes (overall plasma protein glycosylation and concentrations of glycosylated APPs) during inflammation and it has potential to be an ameliorated indicator of inflammation stage and direction after a major surgery, as well as a therapy response marker for an inflammatory treatment.

Despite limitations, our paper is the first (to our knowledge) addressing a topic of great clinical interest. The ability of rating pain and reduce opiates’ consumption have been the cornerstones in improving surgical outcome; as well, biomarkers for pain severity and morphine requirements may lead to personalized targeted approaches to improve patient’s outcome. Thus, our results may pave the way for further studies, since they are the first documented association between a specific biologic marker (i.e. glycome) and a key clinical outcome deeply influencing patients’ recovery.

## Methods

### Plasma/serum samples

#### Discovery cohort

We conducted the study at the Fondazione IRCCS Policlinico San Matteo – Pavia, Italy and at the San Gerardo Hospital - Monza, Italy, after Institutional Review Board-approval from both institutions (06/28/2010, DS2943/2010; 11/11/2010, 611) and registration on ClinicalTrial.gov in November 2010 (NCT01233752). All study subjects provided informed consent. The methods were carried out in accordance with the declaration of Helsinki.

The study has been already described in detail^57^. Plasma was separated by centrifugation for 5 minutes at 4500 rcf, at room temperature. We sent an aliquot of plasma samples of patients enrolled both in Pavia and Monza to Genos.

#### Participants

We enrolled Caucasian patients from January 2011 to July 2013: inclusion criteria were: 18–75 years old, major abdominal surgery (with morphine PCA planned for postoperative analgesia), HIV negative, ASA class I–III. Exclusion criteria were: cognitive or mental impairment, kidney or hepatic failure, preoperative pain, known intolerance to study drugs, postoperative sedation and/or mechanical ventilation in the intensive care. Re-interventions were also excluded from evaluations.

A morphine bolus (0.15 mg/kg ± 20%) was administered 45 min before the end of the surgery, followed by intravenous morphine PCA (1 mg bolus, 5 minutes lock-out, and 20 mg maximal dose every 4 hour) for at least 48 hours. Acetominophen 1 g or ketorolac 30 mg were added at regular intervals.

Samples were collected from each patient through arterial line used as part of intraoperative monitoring; samples were collected at five time points: intraoperative and 6, 12, 24, 48 hours after surgery.

In order to evaluate correlation between morphine consumption and glycomics we analyzed the cumulative distribution of morphine consumption at 24 h. Thus, we calculated the square root of value stratifying patients into three categories: Low (consumption <1 standard deviation of the mean), High (>1 standard deviation of the mean) and Medium (all values in between). Hence, we evaluated differences between baseline and 6-hours glycoproteins level among three groups.

#### Replication cohort

Effects of surgery on the serum N-glycome were evaluated in 28 patients sampled before surgery, 24 h, 48 h and 7 days after surgery, and treated with intravenous morphine PCA. Samples were collected at the Clinical Department for Laboratory Diagnostics at University Hospital Dubrava (Zagreb, Croatia). The study was registered at ClinicalTrials.gov, number NCT01244022 and was approved by the Ethics Committee of University Hospital Dubrava. The methods were carried out in accordance with the declaration of Helsinki. Sera samples were obtained following centrifugation at 1370 × g for 10 minutes in a Rotina 35 R Hettich centrifuge (Tuttlingen, Germany), and then stored at −80 °C until analysis.

#### Participants

The extent of the disease was determined using a standardized protocol. Only patients without evident distant metastases were considered for this study. During the operation, an examination of intraabdominal organs was carried out in search for distant metastases. All resected specimens were subjected to patho-histological examination and in all cases complete local cancer resection (R0 resection) was confirmed.

### Glycan release and labeling

The whole procedure was performed as previously reported^58^. Briefly, the plasma/serum samples were denaturated with the addition of SDS (Invitrogen, USA) and by incubation at 65 °C. The excess of SDS was neutralized with Igepal-CA630 (Sigma-Aldrich, USA) and N-glycans were released following the addition of PNGase F (Promega, USA) in Phosphate Buffer Saline (PBS). The released N-glycans were labeled with 2-AB. Free label and reducing agent were removed from the samples using hydrophilic interaction liquid chromatography solid-phase extraction (HILIC-SPE). Glycans were eluted with ultrapure water and stored at −20 °C until use.

### Ultra-performance liquid chromatography

Fluorescently labeled N-glycans were separated by HILIC on an Acquity UPLC instrument (Waters, USA) consisting of a quaternary solvent manager, sample manager, and an FLR fluorescence detector set with excitation and emission wavelengths of 250 and 428 nm, respectively. The instrument was under the control of Empower 3 software, build 3471 (Waters). Labeled N-glycans were separated on a Waters BEH Glycan chromatography column, 150 × 2.1 mm i.d., 1.7 μm BEH particles, with 100 mM ammonium formate, pH 4.4, as solvent A and ACN as solvent B. The separation method used a linear gradient of 30–47% solvent A at flow rate of 0.56 ml/min in a 23 min analytical run. Samples were maintained at 10 °C before injection, and the separation temperature was 25 °C. Data processing was performed using an automatic processing method with a traditional integration algorithm, after which each chromatogram was manually corrected to maintain the same intervals of integration for all the samples. The chromatograms were all separated in the same manner into 39 peaks as previously reported^58^ for the discovery cohort and for the replication cohort into 37 peaks. For two groups of two chromatographic peaks in the replication cohort we were not able to differentiate between peaks and they were integrated as one measurement (GP12 and GP13 as GP12.13 and GP24 and GP25 as GP24.25). The amount of glycans in each peak was expressed as % of total integrated area.

### Data analysis

Data analysis was conducted with R (version 3.2.2), a free software environment for statistical computing and graphics. Linear mixed effects models (packages lme4^59^ and lmerTest) were used to assess the changes between timepoints (before and after surgery). Reported p-values were adjusted to multiple testing using Benjamini-Hochberg procedure for FDR^60^.

## Additional Information

**How to cite this article**: Gudelj, I. *et al*. Changes in total plasma and serum N-glycome composition and patient-controlled analgesia after major abdominal surgery. *Sci. Rep.* **6**, 31234; doi: 10.1038/srep31234 (2016).

## Supplementary Material

Supplementary Information

## Figures and Tables

**Table 1:** Discovery cohort characteristics.

	Males	Females
N	104	78
Age (years)	64 (23–79)	63 (24–78)
BMI	25.00 (16.81–36.90)	24.56 (17.2–44.86)
Surgery duration (minutes)	197.50 (55–535)	190.00 (50–500)
Type of surgery (%)		
1 upper abdominal	26.9	17.9
2 lower abdominal	20.2	26.9
3 xiphopubic	36.5	44.9
4 bilateral subcostal	16.3	10.3
mgs of morphine first 24 hours	19 (0–95)	17 (0–72)
mgs of morphine between 24 and 48 hours	6 (0–54)	5 (0–34)
Total PCA consumption in 48 h (mgs of morphine)	26 (0–109)	26 (0–88)
Total morphine (including intraoperative bolus)	37 (8, 119)	33 (4–98)

Data are expressed as median (range) unless otherwise stated. BMI = Body Mass Index, PCA = Patient Controlled Analgesia.

**Table 2:** Mean and interquartile range (IQR) measures of glycan levels at intraoperative time point for the discovery cohort together with slope estimates and inference measures (SE-standard error, p value and adjusted p-value) for linear mixed effects models between intraoperative time point and 48 hours after surgery.

Glycan	Mean	IQR	Slope	SE	p	p adjusted
GP1	4.281	2.402	−0.013	0.001	<0.001	<0.001
GP2	1.997	0.817	−0.017	0.002	<0.001	<0.001
GP3	0.123	0.062	−0.017	0.001	<0.001	<0.001
GP4	2.941	1.159	−0.019	0.001	<0.001	<0.001
GP5	1.538	0.69	−0.019	0.001	<0.001	<0.001
GP6	1.107	0.448	−0.017	0.001	<0.001	<0.001
GP7	0.968	0.301	−0.016	0.002	<0.001	<0.001
GP8	1.217	0.374	0	0.002	0.869	1
GP9	0.112	0.045	−0.011	0.002	<0.001	<0.001
GP10	2.743	1.051	−0.017	0.001	<0.001	<0.001
GP11	0.625	0.274	−0.016	0.001	<0.001	<0.001
GP12	1.165	0.253	−0.008	0.001	<0.001	<0.001
GP13	0.607	0.259	−0.015	0.001	<0.001	<0.001
GP14	13.466	2.852	0.006	0.002	<0.001	0.002
GP15	0.566	0.18	−0.008	0.001	<0.001	<0.001
GP16	4.149	1.259	−0.017	0.001	<0.001	<0.001
GP17	1.767	0.799	−0.014	0.001	<0.001	<0.001
GP18	3.593	0.918	−0.002	0.001	0.028	0.278
GP19	0.855	0.273	−0.009	0.002	<0.001	<0.001
GP20	33.538	4.973	0.021	0.001	<0.001	<0.001
GP21	0.741	0.152	0.012	0.001	<0.001	<0.001
GP22	3.907	0.986	−0.001	0.001	0.193	1
GP23	1.82	0.776	−0.014	0.001	<0.001	<0.001
GP24	1.198	0.554	−0.009	0.001	<0.001	<0.001
GP25	0.294	0.106	0.017	0.002	<0.001	<0.001
GP26	1.341	0.447	−0.003	0.001	0.05	0.465
GP27	0.815	0.489	0.009	0.001	<0.001	<0.001
GP28	0.487	0.251	−0.011	0.001	<0.001	<0.001
GP29	0.181	0.107	0.017	0.002	<0.001	<0.001
GP30	4.068	1.734	−0.003	0.001	0.008	0.096
GP31	0.374	0.163	0	0.001	0.774	1
GP32	1.542	0.515	0.004	0.001	0.002	0.031
GP33	2.791	1.587	0.007	0.001	<0.001	<0.001
GP34	0.31	0.129	0.004	0.002	0.006	0.07
GP35	0.32	0.207	0.006	0.001	<0.001	<0.001
GP36	0.368	0.181	0.013	0.002	<0.001	<0.001
GP37	0.308	0.19	0.01	0.002	<0.001	<0.001
GP38	0.748	0.424	0.018	0.002	<0.001	<0.001
GP39	0.722	0.579	0.02	0.001	<0.001	<0.001

**Table 3:** Replication cohort characteristics.

	Male	Female
N	19	9
Age (years)	72 (48–79)	66 (44–85)
Surgery duration (minutes)	130 (94–275)	140 (100–220)
Type of surgery (%)		
1. lower abdominal	64.3	32,2
2. xipho-pubic	3.6	0

**Table 4:** Mean and IQR measures of glycan levels at intraoperative time point for the replication cohort together with slope estimates and inference measures (SE-standard error, p value and adjusted p-value) for linear mixed effects models between intraoperative time point and 48 hours after surgery.

Glycan	Mean	IQR	Slope	SE	p	p adjusted
GP1	5.568	2.485	−0.01	0.001	<0.001	<0.001
GP2	2.438	0.791	−0.017	0.001	<0.001	<0.001
GP3	0.088	0.03	−0.002	0.003	0.414	1
GP4	3.824	1.115	−0.014	0.001	<0.001	<0.001
GP5	1.946	0.981	−0.01	0.001	<0.001	<0.001
GP6	1.436	0.24	−0.018	0.001	<0.001	<0.001
GP7	1.114	0.195	−0.023	0.002	<0.001	<0.001
GP8	0.813	0.21	−0.008	0.002	<0.001	<0.001
GP9	0.085	0.025	−0.009	0.003	0.001	0.007
GP10	3.097	1.18	−0.012	0.001	<0.001	<0.001
GP11	0.792	0.22	−0.013	0.002	<0.001	<0.001
GP12.13	1.766	0.3	−0.021	0.002	<0.001	<0.001
GP14	9.314	1.299	−0.003	0.002	0.122	0.559
GP15	0.432	0.1	−0.006	0.003	0.064	0.301
GP16	4.702	1.355	−0.015	0.001	<0.001	<0.001
GP17	2.127	0.23	−0.012	0.001	<0.001	<0.001
GP18	3.255	0.811	−0.006	0.001	<0.001	<0.001
GP19	0.91	0.205	−0.026	0.002	<0.001	<0.001
GP20	28.643	4.85	0.027	0.002	<0.001	<0.001
GP21	0.631	0.11	−0.002	0.003	0.581	1
GP22	4.386	0.886	0	0.001	0.864	1
GP23	2.652	0.72	−0.013	0.001	<0.001	<0.001
GP24.25	1.66	0.555	−0.01	0.001	<0.001	<0.001
GP26	1.488	0.365	−0.01	0.001	<0.001	<0.001
GP27	0.874	0.375	0.007	0.001	<0.001	<0.001
GP28	0.657	0.28	−0.013	0.001	<0.001	<0.001
GP29	0.168	0.065	0.016	0.002	<0.001	<0.001
GP30	5.155	1.779	−0.004	0.001	<0.001	<0.001
GP31	0.455	0.125	−0.004	0.001	0.001	0.006
GP32	1.854	0.695	0.009	0.001	<0.001	<0.001
GP33	3.916	1.88	0.009	0.001	<0.001	<0.001
GP34	0.387	0.085	0.006	0.002	0.001	0.007
GP35	0.446	0.22	0.006	0.001	<0.001	<0.001
GP36	0.481	0.13	0.019	0.002	<0.001	<0.001
GP37	0.421	0.175	0.015	0.002	<0.001	<0.001
GP38	0.974	0.25	0.026	0.002	<0.001	<0.001
GP39	1.044	0.41	0.023	0.001	<0.001	<0.001
