# Supplementary material for: Changes in total plasma and serum N-glycome composition and patient-controlled analgesia after major abdominal surgery
Source: Sci Rep. 2016 Aug 9;6:31234. doi: 10.1038/srep31234 (PMC4977520; doi:10.1038/srep31234)

Supplementary information file

Changes in total plasma and serum N-glycome composition and patient-controlled analgesia after major abdominal surgery

Ivan Gudelj, Marco Baciarello, Ivo Ugrina, Manuela De Gregori, Valerio Napolioni, Pablo M Ingelmo, Dario Bugada, Simona De Gregori, Lovorka Đerek, Maja Pučić-Baković, Mislav Novokmet, Olga Gornik, Gloria Saccani Jotti, Tiziana Meschi, Gordan Lauc and Massimo Allegri

Supplementary Table S1. Composition of the plasma N-glycome. Plasma N-glycome was separated into 39 chromatographic peaks by HILIC-UPLC.

Glycan peak	Major glycan structure *	General description
GP1	FA2	Agalactosylated
GP2	M5, FA2B	high mannose (M5), agalactosylated with bisecting GlcNAc (FA2B)
GP3	A2[6]BG1	monogalactosylated with bisecting GlcNAc
GP4	FA2[6]G1	monogalactosylated with core fucose
GP5	FA2[3]G1	monogalactosylated with core fucose
GP6	FA2[6]BG1	monogalactosylated with core fucose and bisecting GlcNAc
GP7	M6	high mannose
GP8	A2G2	Digalactosylated
GP9	A2BG2	digalactosylated with bisecting GlcNAc
GP10	FA2G2	digalactosylated with core fucose
GP11	FA2BG2	digalactosylated with core fucose and bisecting GlcNAc
GP12	A2[3]BG1S1	monogalactosylated with bisecting GlcNAc
GP13	FA2[3]G1S1	monogalactosylated and sialylated with core fucose
GP14	A2G2S1	digalactosylated and sialylated
GP15	A2BG2S1	digalactosylated and sialylated with bisecting GlcNAc
GP16	FA2G2S1	digalactosylated and sialylated with core fucose
GP17	FA2BG2S1	digalactosylated and sialylated with bisecting GlcNAc and core fucose
GP18	A2G2S2	digalactosylated and sialylated
GP19	M9	high mannose
GP20	A2G2S2	digalactosylated and sialylated
GP21	A2BG2S2	digalactosylated and sialylated with bisecting GlcNAc
GP22	FA2G2S2	digalactosylated and sialylated with core fucose
GP23	FA2BG2S2	digalactosylated and sialylated with bisecting GlcNAc and core fucose
GP24	A3G3S2	trigalactosylated and sialylated
GP25	A3BG3S2	trigalactosylated and sialylated with bisecting GlcNAc
GP26	A3G3S2	trigalactosylated and sialylated
GP27	A3G3S3	trigalactosylated and sialylated
GP28	A3G3S3	trigalactosylated and sialylated
GP29	FA3G3S3	trigalactosylated and sialylated with core fucose

GP30	A3G3S3	trigalactosylated and sialylated
GP31	FA3G3S3	trigalactosylated and sialylated with core fucose
GP32	A3F1G3S3	trigalactosylated and sialylated with antennary fucose
GP33	A4G4S3	tetragalactosylated and sialylated
GP34	A4G4S3	tetragalactosylated and sialylated
GP35	A4F1G3S3	tetragalactosylated and sialylated with antennary fucose
GP36	A4G4S4	tetragalactosylated and sialylated
GP37	A4G4S4	tetragalactosylated and sialylated
GP38	A4G4S4	tetragalactosylated and sialylated
GP39	A4F1G4S4	tetragalactosylated and sialylated with antennary fucose

** according to Saldova et al., JPR, 2014.*

Supplementary Table S2: Slope estimates and inference measures (SE-standard error, adjusted p values) on the discovery cohort from linear mixed effects models between intraoperative time point and 6, 12, 24 and 48 hours after surgery. Statistically non-significant results (0.05 level) are marked with "-".

	After 6 hours			After 12 hours			After 24 hours			After 48 hours		
glycan	slope	se	p	slope	se	p	slope	se	p	slope	se	p
GP1	-	-	-	-	-	-	-	-	-	-0.013	0.001	<0.001
GP2	0.046	0.014	0.024	-	-	-	-0.014	0.003	<0.001	-0.017	0.002	<0.001
GP3	0.04	0.012	0.02	-	-	-	-0.017	0.003	<0.001	-0.017	0.001	<0.001
GP4	-	-	-	-	-	-	-0.015	0.003	<0.001	-0.019	0.001	<0.001
GP5	-	-	-	-	-	-	-0.016	0.003	<0.001	-0.019	0.001	<0.001
GP6	0.032	0.011	0.048	-	-	-	-0.014	0.002	<0.001	-0.017	0.001	<0.001
GP7	-	-	-	-	-	-	-0.015	0.003	<0.001	-0.016	0.002	<0.001
GP8	-	-	-	-	-	-	-	-	-	-	-	-
GP9	-	-	-	-	-	-	-	-	-	-0.011	0.002	<0.001
GP10	-	-	-	-	-	-	-0.014	0.002	<0.001	-0.017	0.001	<0.001
GP11	-	-	-	-	-	-	-0.018	0.002	<0.001	-0.016	0.001	<0.001
GP12	-	-	-	-	-	-	-	-	-	-0.008	0.001	<0.001
GP13	-	-	-	-	-	-	-0.013	0.003	<0.001	-0.015	0.001	<0.001
GP14	-	-	-	-	-	-	-	-	-	0.006	0.002	0.002
GP15	-	-	-	-	-	-	-	-	-	-0.008	0.001	<0.001
GP16	-	-	-	-	-	-	-0.017	0.002	<0.001	-0.017	0.001	<0.001
GP17	-	-	-	-	-	-	-0.015	0.002	<0.001	-0.014	0.001	<0.001
GP18	-	-	-	-	-	-	-	-	-	-	-	-
GP19	-	-	-	-	-	-	-	-	-	-0.009	0.002	<0.001
GP20	-	-	-	0.024	0.006	0.001	0.031	0.002	<0.001	0.021	0.001	<0.001
GP21	-	-	-	-	-	-	-	-	-	0.012	0.001	<0.001
GP22	-	-	-	-	-	-	-	-	-	-	-	-
GP23	-	-	-	-	-	-	-0.013	0.002	<0.001	-0.014	0.001	<0.001
GP24	-	-	-	-0.021	0.005	0.001	-0.011	0.002	<0.001	-0.009	0.001	<0.001
GP25	-	-	-	-	-	-	0.013	0.004	0.018	0.017	0.002	<0.001
GP26	-0.032	0.011	0.045	-0.029	0.006	<0.001	-	-	-	-	-	-
GP27	-	-	-	-0.021	0.005	0.001	-	-	-	0.009	0.001	<0.001
GP28	-	-	-	-	-	-	-0.007	0.002	0.033	-0.011	0.001	<0.001
GP29	-	-	-	-	-	-	0.021	0.003	<0.001	0.017	0.002	<0.001
GP30	-	-	-	-	-	-	-	-	-	-	-	-
GP31	-	-	-	-	-	-	-	-	-	-	-	-
GP32	-	-	-	-	-	-	-	-	-	0.004	0.001	0.031
GP33	-	-	-	-0.014	0.005	0.045	-	-	-	0.007	0.001	<0.001
GP34	-	-	-	-0.022	0.007	0.018	-	-	-	-	-	-
GP35	-	-	-	-	-	-	-	-	-	0.006	0.001	<0.001
GP36	-0.046	0.014	0.019	-0.024	0.008	0.026	0.012	0.004	0.015	0.013	0.002	<0.001
GP37	-0.041	0.014	0.046	-	-	-	0.012	0.003	0.004	0.01	0.002	<0.001
GP38	-0.05	0.015	0.023	-	-	-	0.02	0.004	<0.001	0.018	0.002	<0.001
GP39	-0.04	0.014	0.049	-	-	-	0.015	0.003	<0.001	0.02	0.001	<0.001

Supplementary Table S3: Slope estimates and inference measures (SE-standard error, adjusted p values) on the replication cohort from linear mixed effects models between intraoperative time point and 24, 48 hours after surgery. Statistically non-significant results (0.05 level) are marked with "-".

	After 24 hours			After 48 hours		
**glycan**	**slope**	**se**	**P**	**Slope**	**se**	**P**
**GP1**	-	-	-	-0.01	0.001	<0.001
**GP2**	-0.011	0.002	<0.001	-0.017	0.001	<0.001
**GP3**	0.019	0.006	0.02	-	-	-
**GP4**	-0.008	0.002	0.003	-0.014	0.001	<0.001
**GP5**	-	-	-	-0.01	0.001	<0.001
**GP6**	-0.011	0.003	0.001	-0.018	0.001	<0.001
**GP7**	-0.013	0.002	<0.001	-0.023	0.002	<0.001
**GP8**	-	-	-	-0.008	0.002	<0.001
**GP9**	0.015	0.005	0.044	-0.009	0.003	0.01
**GP10**	-	-	-	-0.012	0.001	<0.001
**GP11**	-	-	-	-0.013	0.002	<0.001
**GP12.13**	-	-	-	-0.021	0.002	<0.001
**GP14**	-0.013	0.004	0.01	-	-	-
**GP15**	-	-	-	-	-	-
**GP16**	-	-	-	-0.015	0.001	<0.001
**GP17**	-	-	-	-0.012	0.001	<0.001
**GP18**	-0.01	0.001	<0.001	-0.006	0.001	<0.001
**GP19**	-0.018	0.004	<0.001	-0.026	0.002	<0.001
**GP20**	0.021	0.004	<0.001	0.027	0.002	<0.001
**GP21**	-	-	-	-	-	-
**GP22**	-	-	-	-	-	-
**GP23**	-0.008	0.002	0.009	-0.013	0.001	<0.001
**GP24.25**	-0.01	0.002	<0.001	-0.01	0.001	<0.001
**GP26**	-0.011	0.003	0.005	-0.01	0.001	<0.001
**GP27**	-	-	-	0.007	0.001	<0.001
**GP28**	-0.012	0.002	<0.001	-0.013	0.001	<0.001
**GP29**	0.022	0.004	<0.001	0.016	0.002	<0.001
**GP30**	-	-	-	-0.004	0.001	<0.001
**GP31**	-	-	-	-0.004	0.001	0.01
**GP32**	-	-	-	0.009	0.001	<0.001
**GP33**	-	-	-	0.009	0.001	<0.001
**GP34**	-	-	-	0.006	0.002	0.01
**GP35**	-	-	-	0.006	0.001	<0.001
**GP36**	0.022	0.003	<0.001	0.019	0.002	<0.001
**GP37**	0.022	0.003	<0.001	0.015	0.002	<0.001
**GP38**	0.036	0.003	<0.001	0.026	0.002	<0.001
**GP39**	0.02	0.003	<0.001	0.023	0.001	<0.001

Supplementary Table S4: Agreement between estimated slopes for the discovery and the replication cohorts. Same effect between cohorts is marked with green color while the opposite effect is marked with red color. Statistically non-significant results (0.05 level) are marked with "-".

	After 24 hours		After 48 hours	
**glycan**	**Discovery**	**Replication**	**Discovery**	**Replication**
**GP1**	-	-	-0.013	-0.01
**GP2**	-0.014	-0.011	-0.017	-0.017
**GP3**	-0.017	0.019	-0.017	-
**GP4**	-0.015	-0.008	-0.019	-0.014
**GP5**	-0.016	-	-0.019	-0.01
**GP6**	-0.014	-0.011	-0.017	-0.018
**GP7**	-0.015	-0.013	-0.016	-0.023
**GP8**	-	-	-	-0.008
**GP9**	-	0.015	-0.011	-0.009
**GP10**	-0.014	-	-0.017	-0.012
**GP11**	-0.018	-	-0.016	-0.013
**GP14**	-	-0.013	0.006	-
**GP15**	-	-	-0.008	-
**GP16**	-0.017	-	-0.017	-0.015
**GP17**	-0.015	-	-0.014	-0.012
**GP18**	-	-0.01	-	-0.006
**GP19**	-	-0.018	-0.009	-0.026
**GP20**	0.031	0.021	0.021	0.027
**GP21**	-	-	0.012	-
**GP22**	-	-	-	-
**GP23**	-0.013	-0.008	-0.014	-0.013
**GP26**	-	-0.011	-	-0.01
**GP27**	-	-	0.009	0.007
**GP28**	-0.007	-0.012	-0.011	-0.013
**GP29**	0.021	0.022	0.017	0.016
**GP30**	-	-	-	-0.004
**GP31**	-	-	-	-0.004
**GP32**	-	-	0.004	0.009
**GP33**	-	-	0.007	0.009
**GP34**	-	-	-	0.006
**GP35**	-	-	0.006	0.006
**GP36**	0.012	0.022	0.013	0.019
**GP37**	0.012	0.022	0.01	0.015
**GP38**	0.02	0.036	0.018	0.026
**GP39**	0.015	0.02	0.02	0.023

Supplementary Figure S1
Multiple box plots analyzing morphine consumption according to baseline glycoprotein

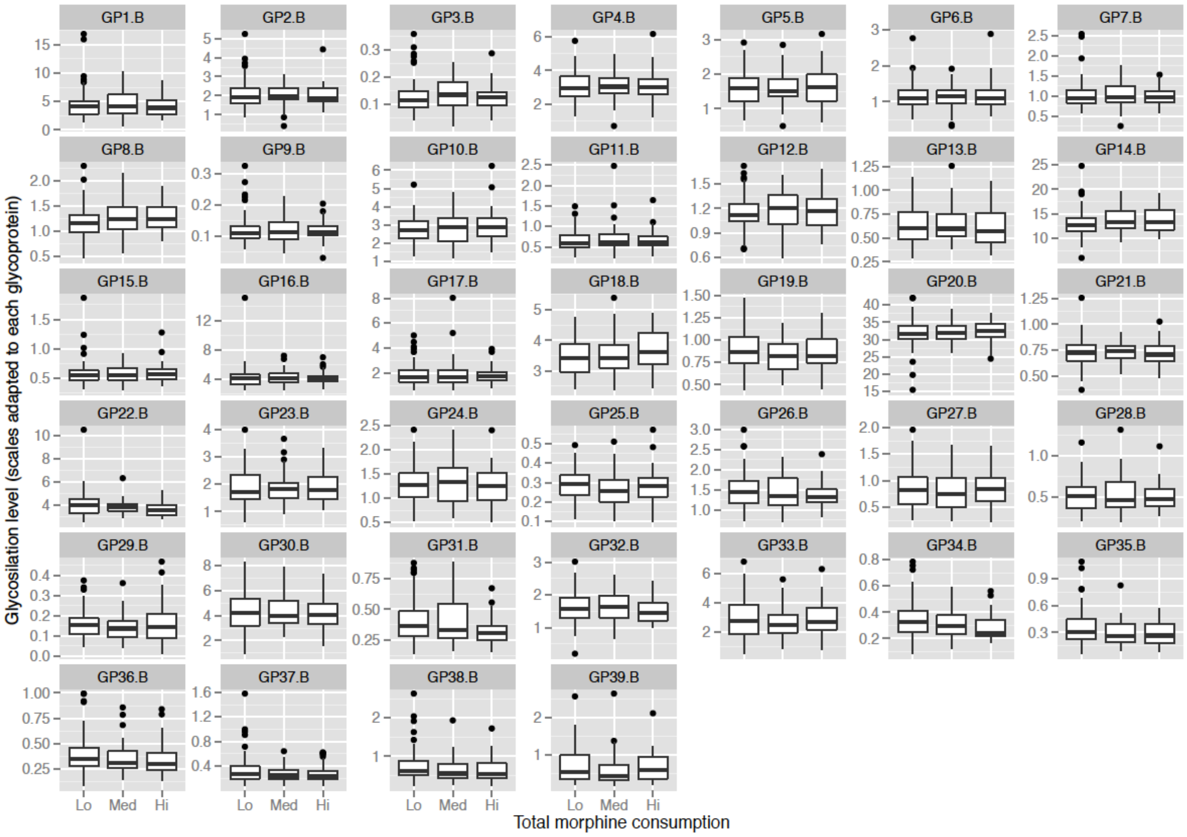

Supplementary Figure S2
Multiple box plots analyzing morphine consumption according to 6-hour glycoprotein levels

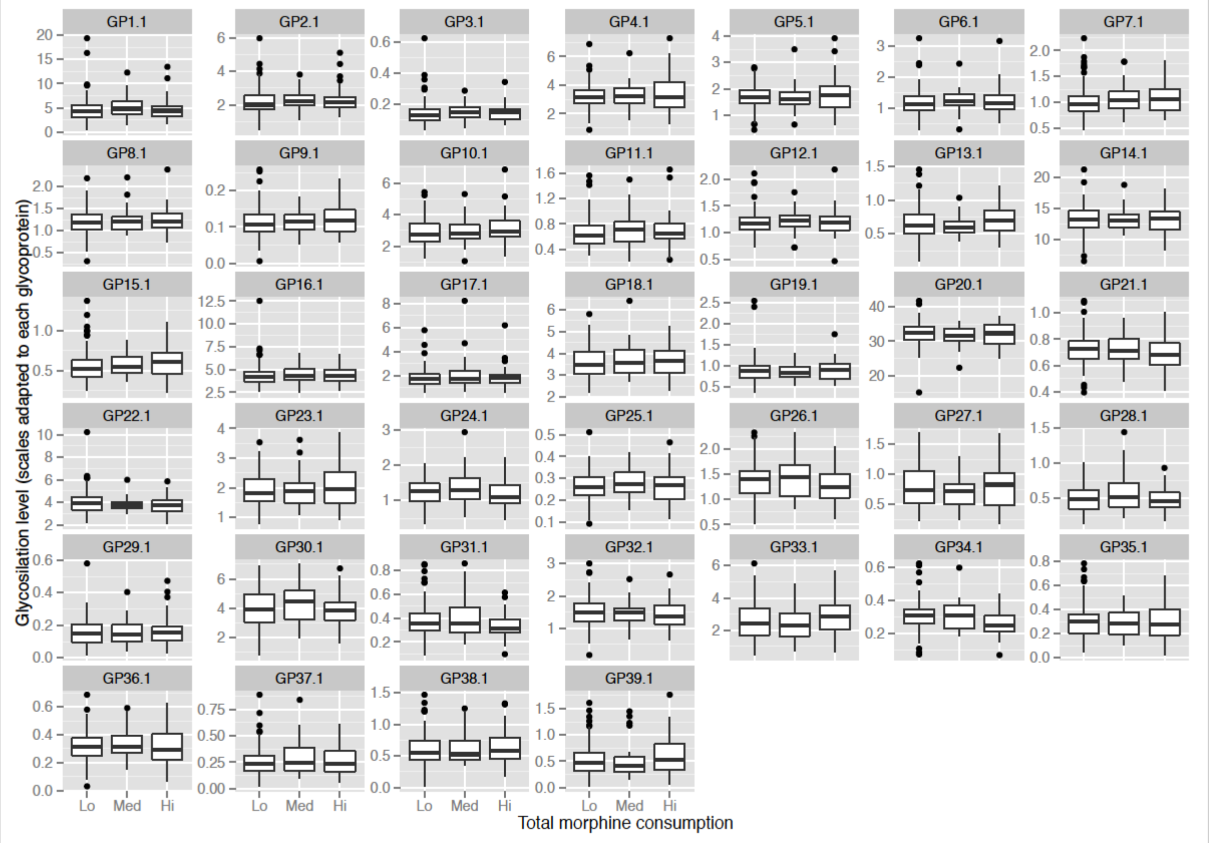

Supplement: Supplementary Information [file srep31234-s1.pdf]
